# Identification of the Expression of TIE1 and Its Mediated Immunosuppression in Gastric Cancer

**DOI:** 10.7150/jca.90891

**Published:** 2024-03-31

**Authors:** Zhenqi Gong, Qing Zheng, Baizhi Li, Huaiming Wang, Hongwu Chen, Shaoxiong Lin

**Affiliations:** 1 Department of Gastrointestinal Surgery, The First Affiliated Hospital of Shantou University Medical College, Shantou, China.; 2 Department of Neurosurgery, The First Affiliated Hospital of Medical College, Shantou University, Shantou, China.; 3 Department of Otolaryngology, The First Affiliated Hospital of Shantou University Medical College, Shantou, China.; 4 Shantou University Medical College, Shantou, China.

**Keywords:** Tyrosine Kinase with Immunoglobulin-like and EGF-like domains 1, Gastric Cancer, Bioinformatics analysis, Immunotherapy, Potential Biomarker.

## Abstract

**Background:** Recently, various evidence has confirmed that Tyrosine Kinase with Immunoglobulin-like and EGF-like domains 1 (TIE1) promotes tumor growth in many cancers. However, the precise mechanism underlying TIE1's involvement in Gastric Cancer (GC) remains elusive. This research aimed to investigate the biological function of TIE1 in regulating GC progression.

**Methods:** The Cancer Genome Atlas (TCGA), Gene Expression Omnibus (GEO), GEPIA2.0, Sangerbox3.0 and TIMER databases were used to analyze the TIE1 expression. Immunohistochemistry (IHC) was used to demonstrate the expression of TIE1. TCGA, GEPIA2.0 and Kaplan-Meier were utilized for survival analysis and to explore the association of TIE1 with clinicopathological features. Protein-Protein Interaction (PPI) networks were constructed using Cytoscape. The potential molecular mechanism of TIE1 was investigated by Gene Ontology (GO), Kyoto Encyclopedia of Gene Genomes (KEGG), and Gene Set Enrichment Analysis (GSEA). We studied the relationships between TIE1 and mutations, immune checkpoints (ICs), tumor mutational burden (TMB), as well as microsatellite instability (MSI) to explore the underlying mechanism of immunity in GC.

**Results:** Compared with normal tissue, TIE1 was significantly overexpressed in GC tissues (p = 0.0072) and was associated with poor survival (P < 0.05). According to GO and KEGG enrichment analyses, TIE1 was enriched in signal pathways related to the occurrence, invasion, and migration of malignant tumors (i.e., PI3K-Akt signaling pathway, Calcium signaling pathway, etc.). Immune infiltration analysis suggested that TIE1 is positively correlated with macrophages M2 and negatively correlated with Mast cells, naive B cells and Follicular helper T cells (TFH), which may be a contributing factor to tumor progression. Furthermore, the research on the tumor microenvironment (TME) and tumor purity also proved that TIE1 may be an oncogene. Mutation analysis showed that the high expression group of TIE1 had a higher frequency of mutations in TP53 and ARID1, while the TMB score was lower.

**Conclusion:** TIE1 might be an oncogene via regulating dysregulated immune infiltration to cause immunosuppression in GC and could be identified as a biomarker for prognosis and a therapeutic target for GC.

## Introduction

Gastric cancer (GC) is a malignant disease with a high incidence and fatality rate worldwide 1, 2. Currently, GC ranks as the fifth most prevalent cancer and has escalated to become the third leading cause of cancer-related deaths globally 3, 4. The highest incidence rates are observed in Asia and Eastern Europe 5. GC often carries a poor prognosis and overall survival due to its common diagnosis at an advanced stage. In 2022, there were approximately 4,820,000 and 2,370,000 new cancer cases, and 3,210,000 and 640,000 cancer deaths in China and the USA, respectively 6. Recent advancements in the field of GC treatment have encompassed various modalities such as surgery, chemotherapy, radiotherapy, and immunotherapy. However, the scarcity of early and accurate diagnostic biomarkers for GC has resulted in a majority of patients facing an adverse prognosis. Consequently, it becomes imperative to identify appropriate prognostic indicators and therapeutic targets that can effectively augment the survival rate of GC patients.

TIE1 (tyrosine kinase with immunoglobulin-like and EGF-like domains 1) is a tyrosine kinase receptor, originally found in an erythroleukemia cell line 7, 8. Previous research has shown that TIE1 is primarily expressed in endothelial cells and plays a crucial role in the formation of blood and lymphatic vessels 9. The mouse tumor model lacking TIE1 reduces tumor angiogenesis and normalizes blood vessels, resulting in increased tumor necrosis and ultimately postponed tumor development 10. TIE1 has been implicated in various cancers including gastric cancer 11, breast cancer 12, cutaneous angiosarcoma 13, colon cancer, etc. 14. Additionally, TIE1 significantly influences the resistance of ovarian cancer cells to platinum therapy 15. However, the exact role of TIE1 in GC and its potential regulatory mechanisms remain uncertain.

In recent years, immunotherapy has emerged as a promising approach, and scientific advancements have expanded our understanding of the immune system's response to malignant cells. Numerous successful immunotherapy methods have been developed, including immune checkpoint inhibitors (ICIs), monoclonal antibodies, cytokines, cellular immunotherapy, and vaccines 16, 17. Additionally, the tumor microenvironment (TME) exerts a profound influence on tumor initiation, progression, invasion, metastasis, and treatment response 18. Recent studies have demonstrated that specific key genes can impact the immune microenvironment of tumors, thereby affecting tumor immunotherapy. For example, UHRF1 in hepatocellular carcinoma (HCC) 19, CXCL11 in colorectal cancer (CRC) 20, AFF3 in GC 21, and so on. In the current study, we observed a significant upregulation of TIE1 in GC which is associated with a poor prognosis for patients. These findings suggest that TIE1 may play a crucial role in the tumorigenesis of GC. Furthermore, tumor-immune infiltrating cells (TIICs) can serve as a valuable prognostic indicator for patients 22. The significant correlation between TIE1 and TIICs indicates its potential as a promising diagnostic biomarker and therapeutic target for GC immunotherapy (See the Graphical Abstract).

## Materials and Methods

### TIE1 gene expression

The mRNA expression level of TIE1 in pan-cancer was determined by using the TIMER database (https://cistrome.shinyapps.io/timer/) and Sangerbox3.0 (http://www.sangerbox.com/home.html). TIMER contains 32 cancer types and employs box plots to visualize the distribution of TIE1 expression levels, and the statistical significance was calculated using the Wilcoxon test. Similarly, there are 10,535 tumor samples from 26 different forms of cancer stored in the Sangerbox3.0 database, which is a component of the integrated and standardized pan-cancer from the Cancer Genome Atlas (TCGA) and Genotype-Tissue Expression databases (GTEx). The TIE1 levels among pan-cancer samples were compared using signed rank and unpaired Wilcoxon rank sum tests. Additionally, four datasets (GSE13911, GSE26899, GSE29272 and GSE54129) acquired from the Gene Expression Omnibus (GEO) database (https://www.ncbi.nlm.nih.gov/geo/) were also used to illustrate TIE1 expression.

### Clinicopathological Characteristics Analysis and Survival Analysis

The transcriptome sequencing and clinical information of GC were acquired from TCGA (https://portal.gdc.cancer.gov). The correlation between clinicopathological characteristics and TIE1 expression was investigated using the “limma” and “survival” packages. Patient clinicopathological features (such as TMN stage and tumor grade) were assessed by conducting a comparative analysis of the high and low expression groups of TIE1. To investigate the potential association between TIE1 expression and GC, we retrieved survival information of GC from TCGA database, and employed Kaplan-Meier curve (https://kmplot.com/analysis/) and GEPIA2.0 database (http://gepia2.cancer-pku.cn/#index) to investigate the impact of TIE1 on GC prognosis, including first-progression survival (FPS), post-progression survival (PPS), receiver operating characteristic (ROC) analysis, and overall survival (OS).

### Correlation analysis and drawing of Protein-Protein Interaction (PPI) network

The Pearson correlation identifies genes exhibiting expression correlation with the TIE1 gene. Based on the screening of highly expressed genes, gene interactions were analyzed using the STRING database (https://cn.string-db.org), and the Cytoscape software was used to construct a PPI network.

### Differentially expressed gene (DEG) functional enrichment analysis

The “limma” package was utilized to stratify TIE1 into high- and low- expression categories based on the gene expression level. The R packages “enrichplot”, “org. Hs.eg.db” and “ggplot2” were employed for conducting functional enrichment analysis of Gene Ontology (GO) and Kyoto Encyclopedia of Gene Genomes (KEGG). GO enrichment analysis was divided into analyses of Cellular Component (CC), Biological Process (BP) and Molecular Function (MF). In addition, we also used “clusterProfiler” and “enrichplot” packages for Gene Set Enrichment Analysis (GSEA). The identification of activities and pathways closely linked to TIE1 was accomplished through functional enrichment analysis.

### The relationship of TIE1 with immune cell tumor infiltration and immune checkpoints

We employed the “limma”, “BiocManager”, “preprocessCore” R packages, along with others, to perform TIE1-related immune cell infiltration analysis. The ESTIMATE algorithm was used to generate the stromal, immune, and estimate scores, which assess the presence of stromal cells and the infiltration of immune cells. This allows researchers to evaluate the correlation between the stromal/immune/estimate score and the expression of TIE1. We utilized data from the TIMER and TISIDB databases (http://cis.hku.hk/TISIDB/index.php) to predict the association between TIE1 expression and tumor immune cells. We investigated the correlation between TIE1 and microsatellite instability (MSI), immune checkpoints (ICs) genes and tumor mutational burden (TMB). Additionally, we also calculated the Immunophenoscore (IPS) to elucidate the impact of TIE1 on immunotherapy.

### Mutation analysis of TIE1

Gene mutation detection is a method of detecting DNA sequence mutations, enabling the prediction of individual disease risk, genetic disease diagnosis, and the development of personalized treatment plans. We obtained somatic mutation data of stomach adenocarcinoma from TCGA and performed a comparative analysis on the somatic mutations between TIE1 high and low expression groups using “maftools” package.

### Immunohistochemical (IHC) analysis of TIE1 expression in GC

We obtained 10 pairs of excised GC tissues and paired normal tissues from clinical practice for IHC staining analysis. The patients were all from the Department of Gastrointestinal Surgery, The First Affiliated Hospital of Shantou University Medical College. The primary antibody was Rabbit Anti-TIE1 antibody (Bioss, bs-1334R), the second antibody was Goat Anti-Rabbit IgG H&L/HRP antibody (Bioss, bs-0295G-HRP), and the DAB kit (AAPR119-A) was from the Pythonbio. We diluted the TIE1 antibody to 1:100 according to the instructions provided in the product manual. The stained paraffin sections were imaged with the microscope (200× magnified), and the cytoplasm stained in brownish yellow color was considered positive. ImageJ software was used to analyze the IHC staining results and calculate the OD value of the positive area. Statistical analysis was performed using t-test, and finally, a bar chart depicting these results was created using GraphPad Prism software.

### Statistical analysis

R program was used for statistical analysis (version 4.3.1). Cytoscape software (version 3.10.0) and GraphPad Prism (version 9.5.1) were used for drawing. The Wilcoxon test was used to compare the levels of TIE1 expression across groups of tissues. T-test was used to analyze IHC results. Kaplan-Meier curves demonstrated how TIE1's differential expression impacts GC survival. We Obtained correlation genes using Pearson correlation analysis (Cor > 0.6, P-value < 0.05). Differential expression analysis was performed based on high expression and low expression groups of the TIE1 gene, and FDR (false discovery rate) < 0.05 and |log2FoldChange| > 1 were selected as the threshold for screening for DEGs. P < 0.05 was considered meaningful and significant.

## Results

### Expression level of TIE1

**Figure 1:**
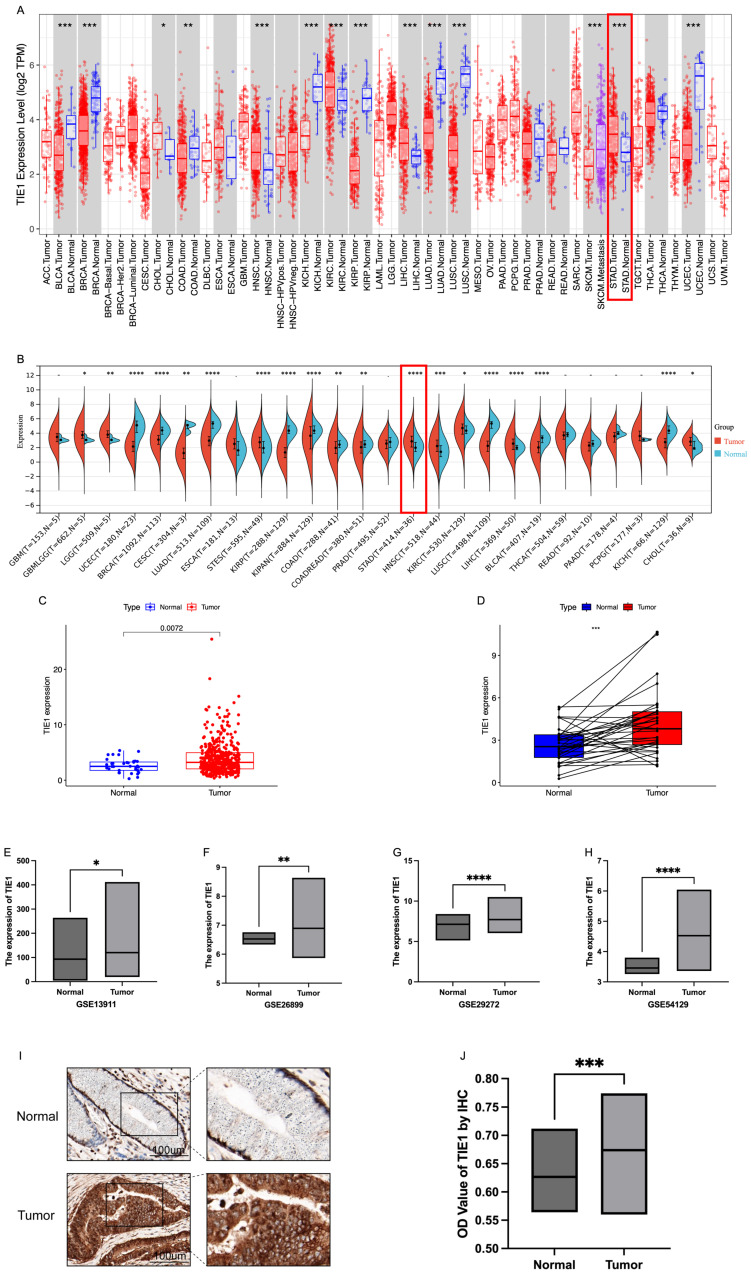
** Expression level of TIE1.** (A) TIE1 expression in pan-cancer in TIMER database. (B) TIE1 expression in pan-cancer in Sangerbox3.0 database. (C) TIE1 expression between 412 tumor tissues and 36 normal tissues in GC by using TCGA RNA-seq data. (D) Paired difference analysis of TIE1 expression. (E-H) TIE1 expression in (E) GSE13911, (F) GSE26899, (G) GSE29272 and (H) GSE54129 datasets from the GEO database. (I, J) IHC analysis of TIE1 expression in GC. TIE1 expression is upregulated in GC tissues compared to adjacent normal tissues. (I) The protein expression level of TIE1 was detected in 10 pairs of GC and adjacent normal tissues via IHC staining (200×). (J) The column result of IHC staining. (*P < 0.05, **P < 0.01, ***P < 0.001.)

The expression profile of TIE1 exhibits considerable heterogeneity across various tumor types. In this study, we investigated the expression of TIE1 in pan-cancer using the TIMER database. Our analysis revealed that TIE1 is downregulated compared to normal tissues in bladder carcinoma (BLCA), breast cancer (BRCA), kidney chromophobe (KICH), kidney renal papillary cell carcinoma (KIRP), lung adenocarcinoma (LUAD), lung squamous cell carcinoma (LUSC) and uterine corpus endometrial carcinoma (UCEC). Conversely, higher levels of TIE1 were observed in cholangiocarcinoma (CHOL), head and neck squamous cell carcinoma (HNSC), kidney renal clear cell carcinoma (KIRC), liver hepatocellular carcinoma (LIHC) and stomach adenocarcinoma (STAD) compared to normal tissues (Figure 1A). These findings are further supported by data from the Sangerbox3.0 database (Figure 1B).

In TIMER and Sangerbox3.0 databases, in contrast to normal tissues, a significant upregulation of TIE1 expression in GC was observed compared to normal tissues (P < 0.05). To further validate this finding, we analyzed RNA-seq data from TCGA, which included 412 GC tumor tissues and 36 normal tissues. Consistently, our analysis confirmed the above reported elevated expression of TIE1 in GC (Figure 1C). Additionally, paired difference analysis yielded the same result (Figure 1D). Next, we obtained GSE13911, GSE26899, GSE29272 and GSE54129 datasets from the GEO database for validation, and these datasets also demonstrated high expression levels of TIE1 in GC (Figures 1E-H). Collectively, these findings strongly support the notion that TIE1 serves as a critical regulatory factor in various cancers including GC, and it is highly expressed in GC.

Next, we evaluated the expression of TIE1 at the organizational level by measuring its expression level in 10 GC patients for IHC analysis. Randomly, we selected 5 fields of view for each sample to calculate the OD value of the positive area and compared them (Table S1). We found that TIE1 expression was higher in GC than in paired adjacent normal tissues (Figures 1I, 1J). The results are consistent with the results analyzed from the database.

### TIE1 expression is correlated with clinical parameters in GC

**Figure 2:**
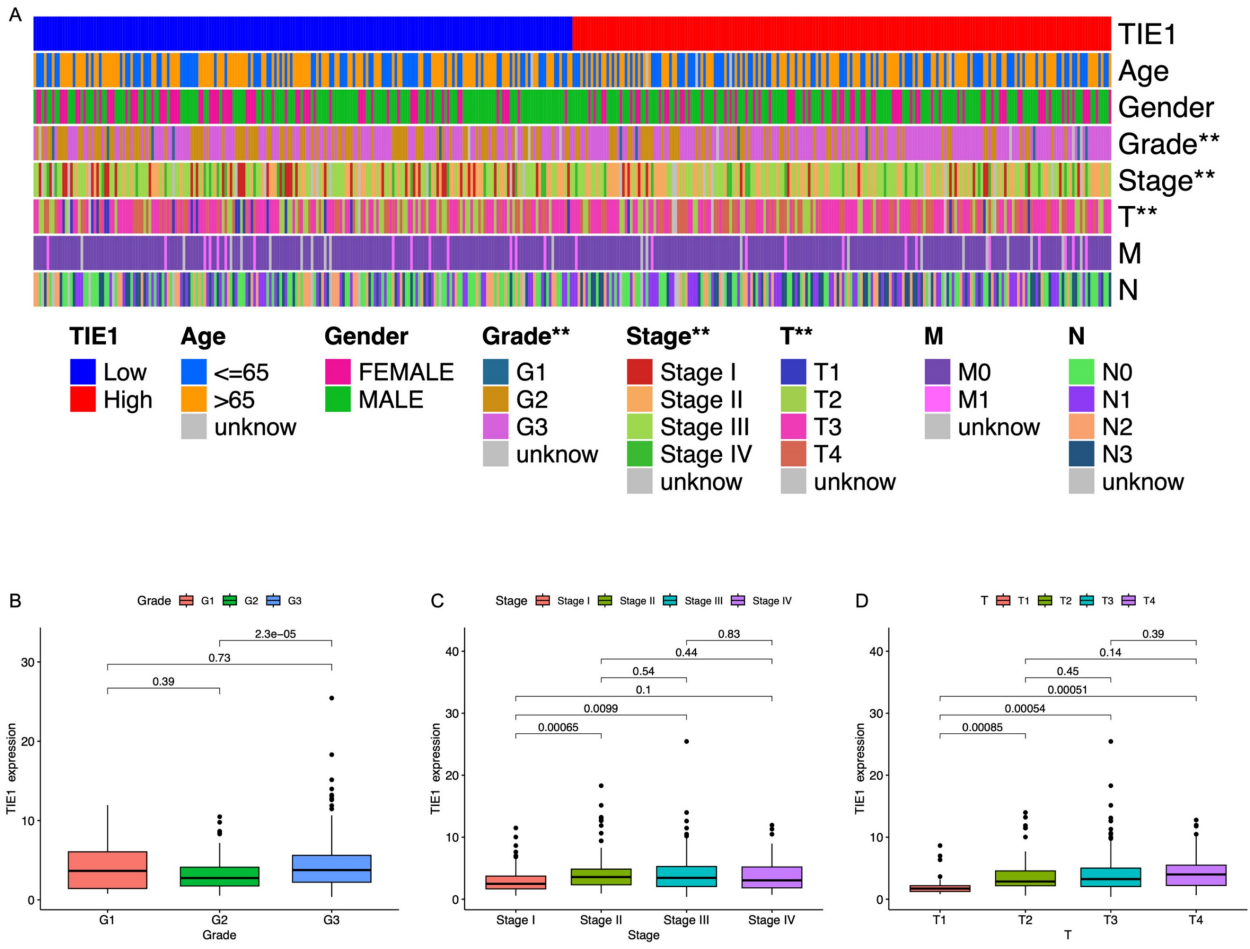
** TIE1 expression is correlated with clinical parameters in GC.** (A) Heatmap of correlation between TIE1 expression and clinical factors. (B-D) The relationship between TIE1 with (B) grade, (C) stage and (D) T staging of GC.

To investigate the role of TIE1 in GC, we examined its association with clinical characteristics. Utilizing RNA-seq data obtained from TCGA, GC patients were categorized based on age, sex, grade, stage, tumor size, lymph node metastasis, and distant metastasis (Table S2). Our analysis revealed a significant correlation between TIE1 expression and GC grading as well as T staging. However, no substantial associations were observed about age, gender, or M/N stages (Figure 2A). The study demonstrated a statistically significant difference in TIE1 expression between grade III and grade II groups, indicating an exceptionally high concentration of TIE1 within the grade III group (Figure 2B). In terms of tumor stage, while no significant difference was observed in the expression of TIE1 among stage II, III, and IV groups, the expression of TIE1 in the stage I group was significantly lower than that in stage II, III and IV groups (Figure 2C). Similarly, we observed a significant downregulation of TIE1 expression in the T1 group compared to the T2, T3, and T4 groups (Figure 2D). According to these findings, there is a strong association between elevated levels of TIE1 expression and unfavorable clinical characteristics in GC.

### TIE1 expression is related to prognosis in GC

**Figure 3:**
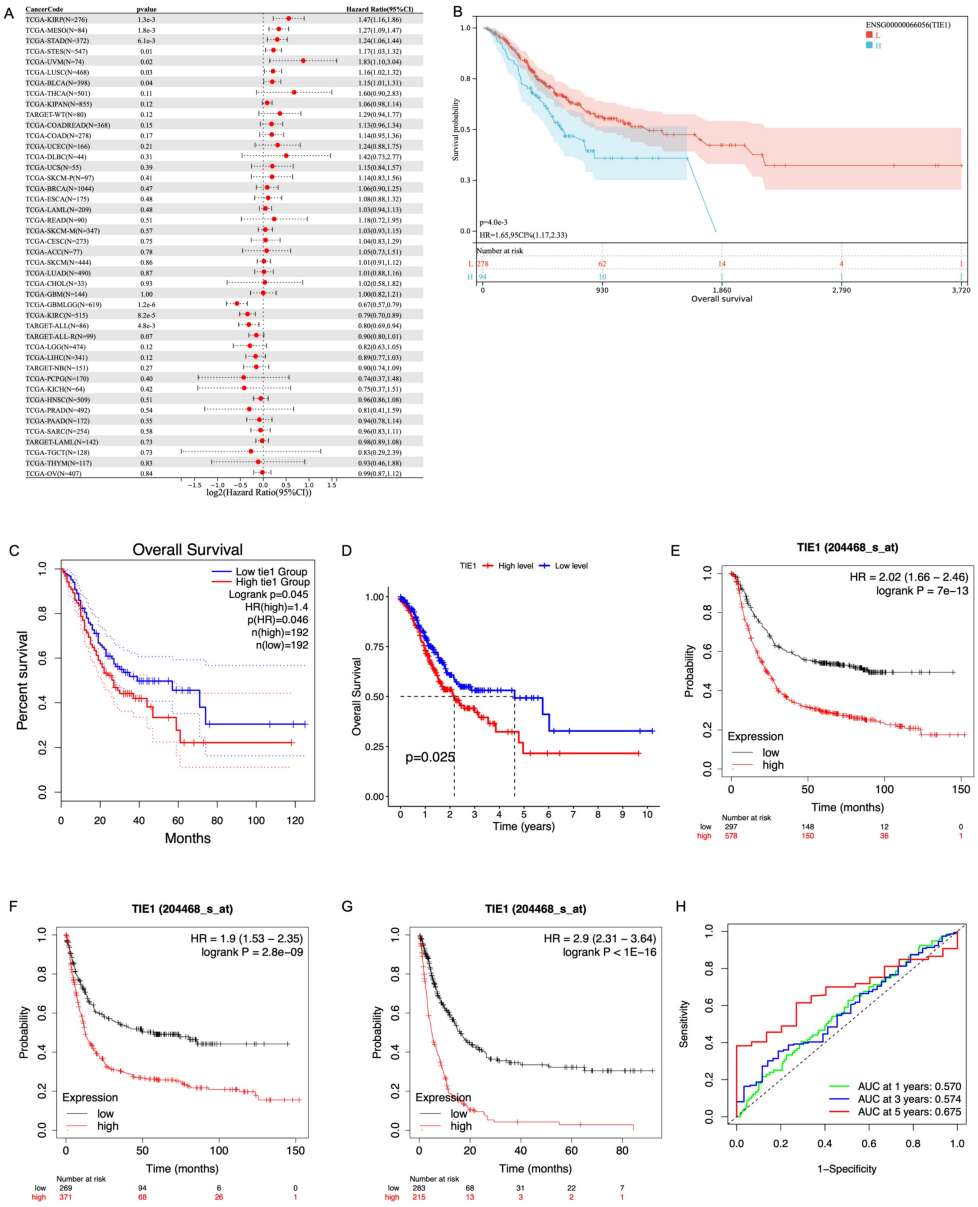
** TIE1 expression is related to prognosis in GC.** (A, B) The relationship between the expression of TIE1 and OS in (A) pan-cancer and (B) STAD from Sangerbox3.0 database. (C, D) OS between high level and low level of TIE1 by (C) GEPIA2.0 and (D) R analysis. (E-G) The Kaplan-Meier curves for (E) OS, (F) FPS and (G) PPS of TIE1. (H) ROC curve to display the 1, 3, and 5-year AUC of TIE1.

Based on the median expression of TIE1, we stratified the patients into a high-expression group and a low-expression group. Subsequently, we used clinical data obtained from the TCGA database to evaluate the predictive value of TIE1. Firstly, we investigated the relationship between TIE1 expression and OS by utilizing the Sangerbox3.0 database. Our analysis revealed that in STAD, elevated levels of TIE1 expression are associated with a significantly reduced survival rate (p = 4.0e-3) (Figures 3A, B). GEPIA2.0 and the outcomes of R analysis showed the same results (Figures 3C, D). Then, we utilized the Kaplan-Meier tool to construct the correlation curves for OS, FPS and PPS (Figures 3E, F, G). In addition, we further generated the ROC curve and calculated the corresponding area under the curve (AUC) in the first, third and fifth years, yielding values of 0.570, 0.574, and 0.675 respectively (Figure 3H). The current findings suggest that the expression of TIE1 holds promise as a prognostic indicator for GC.

### Genetic correlation analysis and drawing of Protein-Protein Interaction (PPI) network

**Figure 4:**
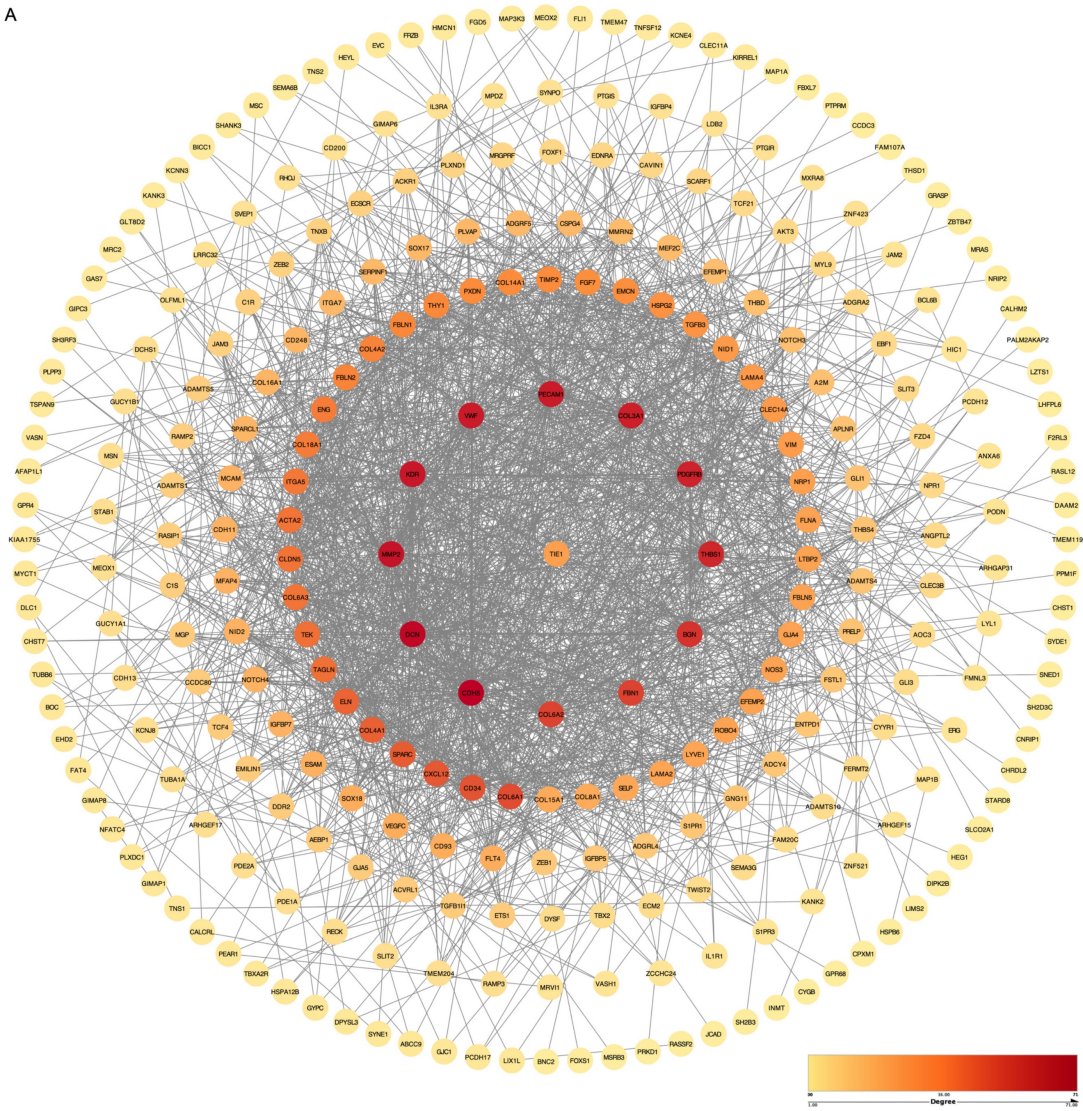
** Genetic correlation analysis and drawing of Protein-Protein Interaction (PPI) network.** (A) PPI network of TIE1. (The order of interaction intensity: Crimson >Orange >Deep yellow >Medium yellow >Light yellow.)

To comprehensively investigate the PPI in biological systems, we conducted a systematic analysis of all genes to determine their correlation with the TIE1 gene using the Pearson correlation method. Remarkably, our findings revealed a significantly positive correlation (cor > 0.6, p-value < 0.05) between the TIE1 gene and 340 other genes. Subsequently, we imported 340 related genes along with TIE1 into the STRING database to elucidate their interaction relationships and construct a PPI network using Cytoscape, which includes 288 nodes and 1953 edges (Figures 4A). Notably, darker colors in the figure indicate stronger correlations among proteins. Consequently, our analyses identified significant correlations between TIE1 and CDH5, KDR, MMP2 among others, moreover, these genes were found to be highly expressed in GC and associated with unfavorable prognosis 23-25.

### Functional and Pathway Enrichment Analysis of TIE1

**Figure 5:**
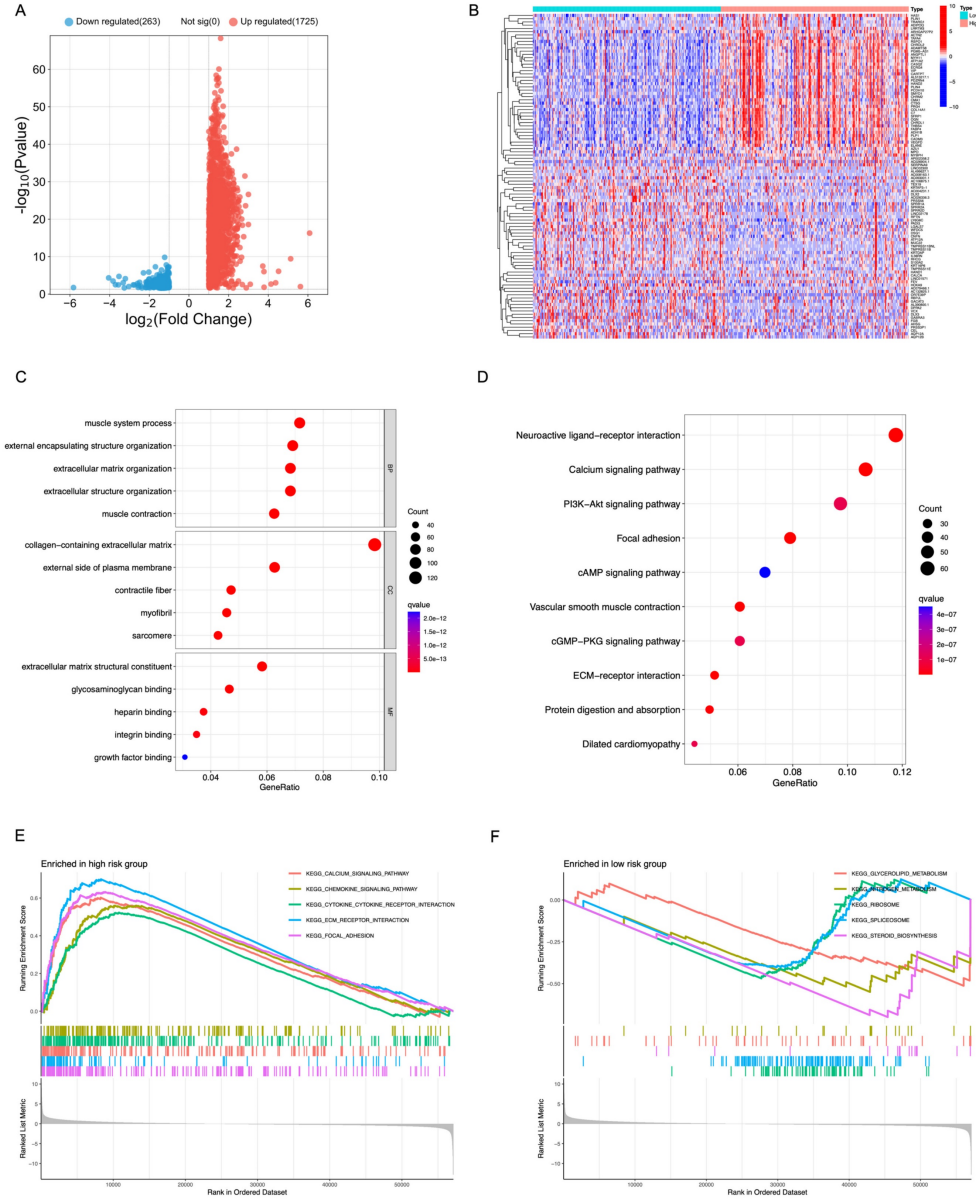
** Functional and Pathway Enrichment Analysis of TIE1.** (A) Volcano plot representing the differentially expressed between the up and the down groups. (B) A heatmap of the top 50 genes that were upregulated and downregulated separately. (C, D) Bubble chart of (C) GO analysis and (D) KEGG analysis. (E, F) GSEA analysis of (E) the high expression group and (F) the low expression group of TIE1.

In order to assess the biological role of TIE1 in GC, we identified 1988 DEGs from the TCGA dataset. Among these DEGs, 1725 genes were upregulated while 263 genes were downregulated, and we visually represented these findings through a volcano plot (Figure 5A).

Furthermore, we separately filtered the top 50 upregulated and downregulated genes to construct a heatmap for better visualization (Figure 5B). Additionally, functional enrichment analyses using GO and KEGG databases revealed that the DEGs were primarily associated with processes related to muscle system process, collagen-containing extracellular matrix and extracellular matrix structural constituent (Figure 5C). Pathway analysis based on KEGG database demonstrated that these DEGs predominantly regulate pathways such as Calcium signaling pathway, PI3K-Akt signal pathway, Focal adhesion and cAMP signaling pathway (Figure 5D).

GSEA analysis was performed to compare the high-expression group of TIE1 gene with the low-expression group, revealing significant pathways. Among them, the high-expression group exhibited a significant enrichment of 52 KEGG pathways, with the top 5 being prominently displayed (Figure 5E). Conversely, the low-expression group demonstrated a significant enrichment of 7 KEGG pathways and also displayed the top 5 (Figure 5F). All these results indicate a potential mechanistic pathway through which TIE1 may promote tumor progression.

### TIE1 expression is related to the infiltration of immune cells

**Figure 6:**
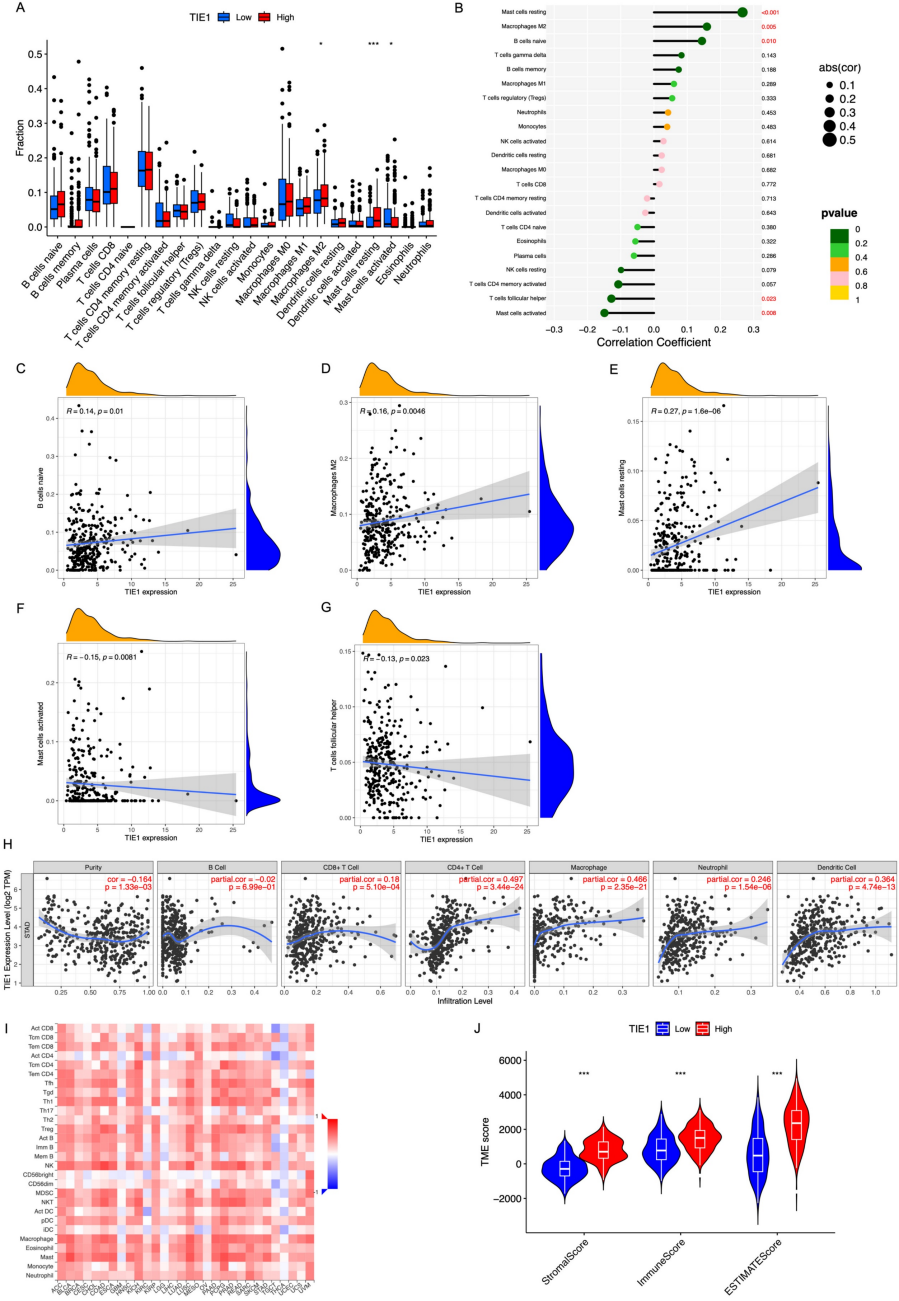
** TIE1 expression is related to the infiltration of immune cells.** (A) Box plot of immune cell infiltration of TIE1. (B) Lollipop diagram of the relationship between immune cells and TIE1 expression. (C-G) The relationship between the expression level of TIE1 with (C) B cells naive, (D) Macrophages M2, (E) Mast cells resting, (F) Mast cells activated and (G) T cells follicular helper. (H, I) The relationship between the expression level of TIE1 and the abundance of TIICs by (H) TIMER and (I) TISIDB. (J) Violin of the relationship between the ESTIMATE score and the expression of TIE1.

To gain further insights into the potential mechanism of TIE1 in GC, we analyzed the relationship between TIE1 and immune cell infiltration. Utilizing CIBERSOFT to process data from the TCGA database, our findings revealed that high expression of TIE1 was associated with increased levels of macrophages M2 cells and decreased levels of activated mast cells (Figure 6A). Specifically, we observed a positive correlation between TIE1 and various cell types, including B cells naive (R = 0.14, p = 0.01), Macrophages M2 (R = 0.16, p = 0.0046), Mast cells resting (R = 0.27, p = 1.6e-06). However, there was a negative correlation between TIE1 and Mast cells activated (R = -0.15, p = 0.0081) as well as T cells follicular helper (R = -0.13 p = 0.023) (Figures 6B-G). These results mean that overexpression of TIE1 may cause infiltration of immunosuppressive cells and contribute to cancer cell metastasis in GC.

TIICs play a role in anti-tumor immune response in the host, thereby influencing tumor occurrence and spread. TIICs encompass various cell types such as CD4^+^T cells, CD8^+^T cells, B cells, Macrophages, etc., which play different roles. In order to better understand the correlation between TIE1 expression levels and TIIC abundance, we conducted an investigation utilizing the TIMER database. Our analysis revealed a robust negative association between tumor purity and TIE1 (r = -0.164 p = 1.33e-03) (Figure 6H), which was consistent with findings from TISIDB (Figure 6I). These results further validated that the abundance of TIICs in TME may be significantly influenced by the level of TIE1.

In addition, TME is regulated by both adaptive and innate immune cell infiltration. Therefore, we employed ESTIMATE analysis to evaluate the relationship between TIE1 expression and TME score to assess the impact of TIE1 on TME and its influence on tumor prognosis. The results indicated that the Stromal/Immune/Estimation Scores were significantly higher in the high-expression groups of TIE1 compared to those with low-expression (Figure 6J). This means that there may be more immune cell infiltration in the high expression group of TIE1, but most of the infiltrating immune cells are immunosuppressive cells, which is unfavorable for the survival prognosis of GC patients.

### Relationship between the TIE1 expression with the immune checkpoints and the Effect of immunotherapy

**Figure 7:**
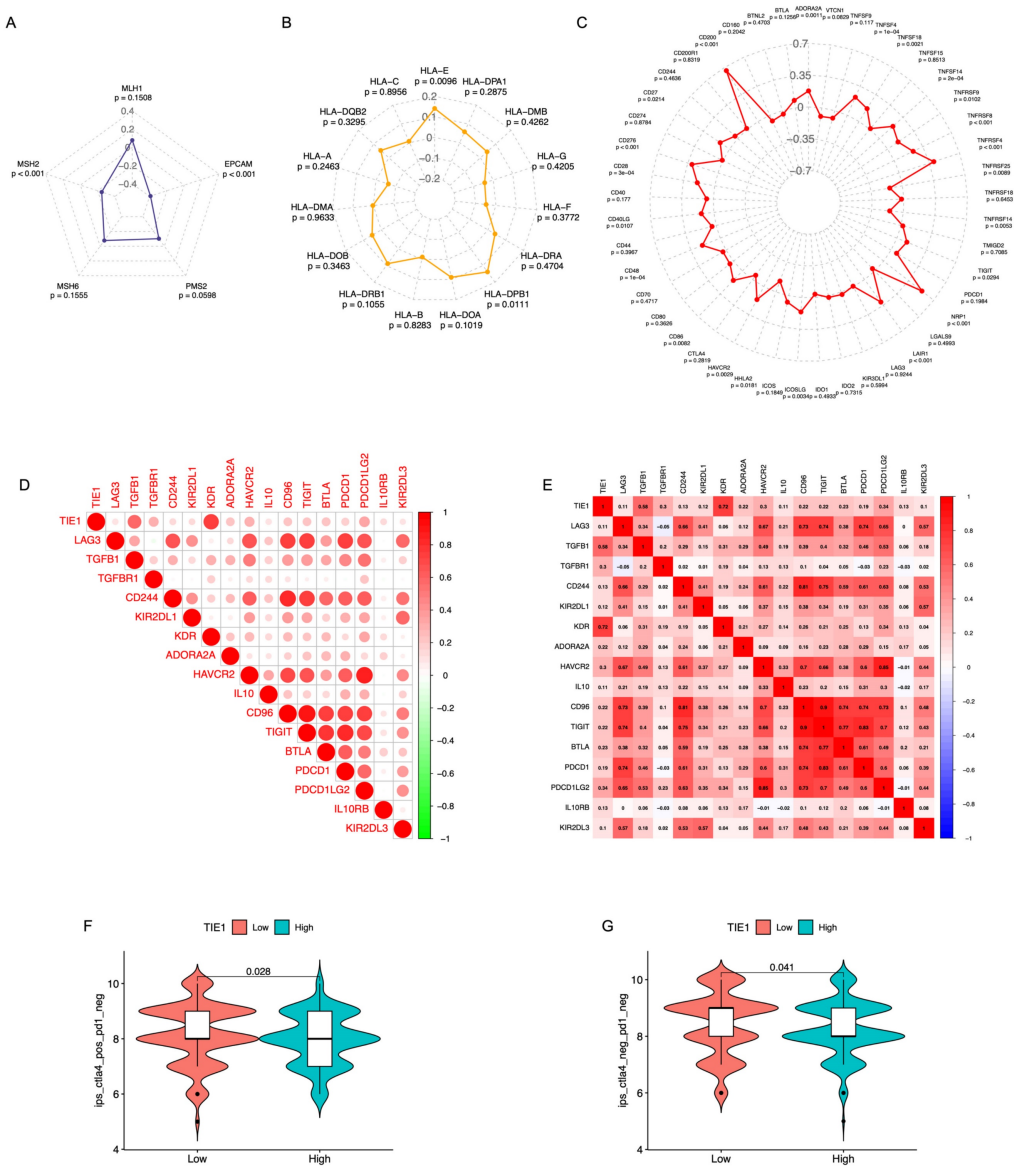
**Relationship between the TIE1 expression with the immune checkpoints and the Effect of immunotherapy.** (A-C) Radar chart of the correlation between TIE1 gene and (A) MMR genes, (B) HLA family genes and (C) ICs genes. (D) The relationship between 17 immune checkpoints with TIE1 expression levels. (E) Quantitative analysis of the correlation between TIE1 expression level and immune checkpoint. (F, G) The expression of TIE1 and immunotherapy.

Pearson correlation analysis was performed to examine the relationship between the TIE1 gene and 5 MMR genes, 15 HLA family genes, as well as 45 ICs genes, subsequently, 3 radar charts were plotted (Figures 7A-C). We found there is a significant association between TIE1 expression and the expression of select MMR genes, HLA genes, and ICs genes. Then, to evaluate the value of TIE1 in GC immunotherapy, we investigated the relationship between TIE1 expression levels and ICs in GC and discovered a positive association between TIE1 expression levels and 17 ICs, thereby highlighting their potential relevance in the context of GC immunotherapy. Furthermore, we generated a comparable heatmap and conducted a quantitative analysis to explore the relationship between ICs and TIE1 expression level (Figures 7D, 7E). Notably, significant positive correlations were observed for key ICs including PDCD1, PDCD1LG2, TGFBR1, IL-10, and ADORA2A (adenosine A2a receptor). Additionally, we calculated the IPS and further analyzed the association between TIE1 expression and immunotherapy. Interestingly, we found a negative correlation between TIE1 expression and CTLA4^+^/PD1^-^ as well as CTLA4^-^/PD1^-^ (Figures 7F, 7G). Collectively, these findings imply that TIE1 may influence the response to immunotherapy.

### Mutation Analysis

**Figure 8:**
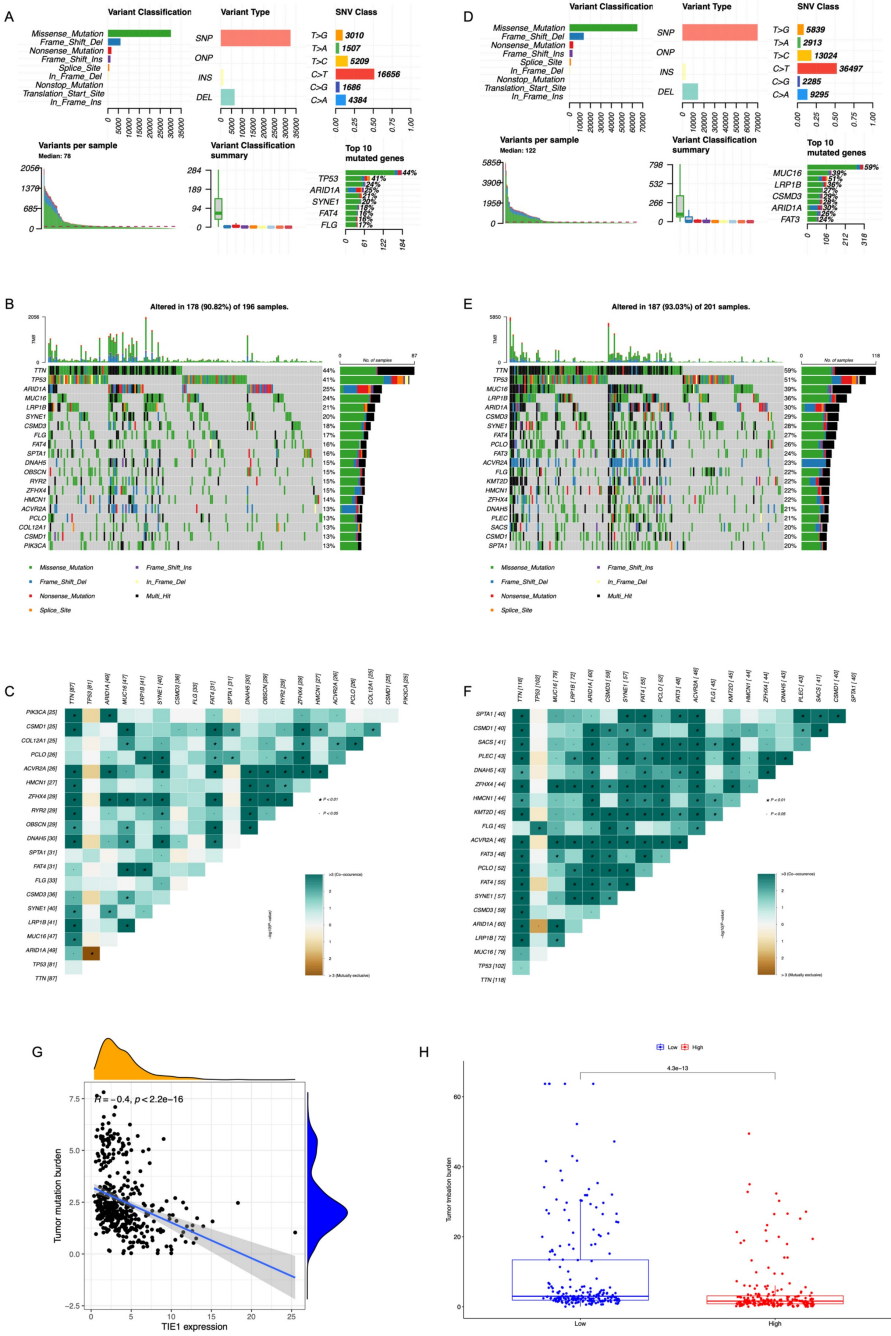
** Mutation Analysis.** Overview of mutation analysis of (A) TIE1 high expression group and (D) TIE1 low expression group. A waterfall graph of the top 20 mutated genes of (B) TIE1 high expression group and (E) TIE1 low expression group. The correlation analysis of the top 20 mutated genes of (C) TIE1 high expression group and (F) TIE1 low expression group. (G) The relationship between TIE1 expression and TMB. (H) TMB differences between high and low expression groups of TIE1.

Based on the GC cell mutation data from TCGA, we compared the differences in the top 20 gene mutations between the groups with high and low expression levels of TIE1. We could see that in the high expression group, Missense Mutation was the main type of mutation, and TP53, ARID1A, SYNE1, FAT4, and FLG were among the first few mutated genes (Figure 8A). Additionally, we plotted a waterfall graph of the top 20 mutated genes according to their mutation types and conducted correlation analysis (Figures 8B, 8C). Similarly, in the low expression group, Missense Mutation was also the main type of mutation, and the first few mutated genes were MUC16, LRP1B, CSMD3, ARID1A, and FAT3 (Figure 8D). Next, a waterfall chart was also drawn and a correlation analysis was conducted (Figures 8E, 8F). TP53 is the most important tumor suppressor gene in the human body. Mutations in TP53 can lose their tumor suppressor activity, which may lead to the development of tumors. This could be one possible explanation for why TIE1 promotes the progression of GC.

We also conducted a comparative analysis of TMB between the high and low expression groups (Figure 8G). According to the findings, there was a strong correlation between TMB and TIE1 expression, particularly exhibiting a negative association with STAD (R = -0.4, p < 2.2e-16) (Figure 8H). Additionally, increased TIE1 expression is associated with lower TMB, potentially leading to weaker immune responses.

## Discussion

Due to its high incidence rate and rapid development, GC poses a significant challenge in achieving early diagnosis using current examination methods, resulting in a substantial mortality rate. The clinical efficacy of conventional therapy remains limited, along with the availability of effective early-diagnostic methods 17. Most cases of GC are diagnosed at an advanced stage after the tumor cells have spread to distant organs, and the median overall survival time (mOS) of advanced GC is only about 8 months 26. Currently, there exists some markers for the prediction of GC, however, the number of immune-related targets and markers remains limited, and the immune mechanism is still unclear. Therefore, identifying appropriate prognostic markers and prolonging patient survival through immunotherapy poses a great challenge. TIE1 is a tyrosine kinase receptor in endothelial cells, where it regulates Angiopoietin/TIE2 signaling 27. TIE1 potentially influences cardiac development, restricts the formation of blood vessels during venous system development, and actively engages in lymph angiogenesis among other processes 27-29. More and more literature has demonstrated aberrant expression of TIE1 in diverse tumor types, with several studies indicating its potential role in promoting carcinogenesis and tumor progression. For example, breast cancer, colorectal cancer, nasopharyngeal carcinoma, to name a few 8, 12, 30.

In this study, evidence from multiple databases revealed that TIE1 exhibited significant overexpression in various cancer types, including GC. And in GC tissue, we also confirmed an increase in the expression of TIE1. Moreover, elevated TIE1 expression in GC was consistently associated with unfavorable clinicopathological features. Kaplan-Meier survival analysis demonstrated that patients with higher levels of TIE1 expression in GC experienced shorter overall survival time and poorer quality. Mechanistically, it is plausible that TIE1 may mediate PI3K-Akt signal pathway to promote the progression of GC. These findings point to a possible tumor-promoting role for TIE1 and highlight the strong correlation between TIE1 expression levels and the prognosis of GC patients, positioning this gene as a promising candidate for utilization as a GC biomarker.

Immunotherapy represents a widely employed therapeutic approach at present. The efficacy of immunotherapy usually depends on the interaction of immune regulation in TME 31. The TME (including various types of lymphocytes and tumor related macrophages, TAMs), comprising diverse cell types such as stromal cells, endothelial cells, fibroblasts, and immunological cells, plays a crucial role in the generation and survival of tumor cells 32. Tumor purity refers to the proportion of tumor cells in the tumor to all cells, a higher abundance of stromal and immune cells indicates lower tumor purity, and vice versa. The TME exerts a profound impact on the efficacy of cancer immunotherapy. It has been suggested that the inclusion of immune cells and stromal cells, which are crucial non-tumor constituents within the TME, may offer valuable insights for both diagnostic purposes and prognostic evaluation in patients with tumors. The ESTIMATE algorithm-based immune score and stromal score facilitate tumor quantification by effectively distinguishing between immune and stromal components 33. This method accurately predicts the infiltration of non-tumor cells by using unique gene expression features specific to immune and stromal cells. The use of ESTIMATE analysis to reflect the abundance of immune cells and stromal cells in tumor samples can be used to evaluate immune cell infiltration and immune response levels. Consequently, an elevated immune/stromal/estimate score coupled with reduced tumor purity independently serves as a risk factor for a poor overall survival rate in GC. In our study, we observed a favorable association between high immune/stromal/estimate score and low tumor purity with TIE1 overexpression, indicating that immune cell infiltration was more frequent in the TIE1 high expression group. Based on further analysis of immune cell infiltration, we found that the infiltrating cells were mostly immunosuppressive cells, which may be the reason for the poor prognosis of GC patients in the TIE1 high expression group.

To investigate the potential correlation between TIE1 expression and the immune microenvironment of GC, we obtained the relevant data from the TCGA database. Our analysis revealed that the expression of TIE1 is related to different levels of immune cell infiltration in GC. The CIBERSORT analysis showed a positive correlation between the high expression of TIE1 and the infiltration levels of Macrophages M2, Mast cells resting and B cells naive. Conversely, TIE1 was negatively correlated with Mast cells activated and T cells follicular helper. Moreover, we conducted comprehensive analyses utilizing the TIMER database to elucidate the potential association between elevated TIE1 expression and immunosuppressive cell infiltration levels. Previous studies have shown that infiltration of naive B cells in GC is associated with an unfavorable prognosis 34. It is widely acknowledged that excessive activation of M2 macrophages can contribute to fibrosis during tissue healing and possibly encourage tumor development via immunosuppression 35. In this research, we investigated the positive correlation between the expression of naive B cells and TIE1, and we further observed higher levels of M2 macrophages and lower levels of M1 macrophages in GC patients with elevated TIE1 expression. In addition, our findings have revealed that elevated TIE1 expression in GC patients is associated with reduced levels of Follicular helper T (TFH) cells. It is noteworthy that TFH cells can promote the formation of tertiary lymphatic structures, enhance tumor immune infiltration, and inhibit tumor growth. So, this observation has garnered significant attention in recent years 36. To sum up, our results suggest that the high expression of TIE1 may encourage the infiltration of dysregulated immune cells, which sheds light on the underlying link between TIE1 expression and GC prognosis.

In the immune system, immune checkpoint molecules 37 are recognized as pivotal regulators of T lymphocyte activation, playing a crucial function in maintaining immunological balance and preventing excessive immune response. These molecules function as essential negative modulators that effectively control the stimulation of T cells 38, thereby safeguarding immune homeostasis and averting hyperactivation of the immune system. Programmed cell death 1 (PD1) and Cytotoxic T lymphocyte antigen 4 (CTLA4) are the most concerned and effective T-cell immune checkpoint molecules 39. While ICIs therapy has shown benefits for certain individuals with metastatic malignancies, there remains a need for prognostic biomarkers. TMB may potentially serve as a predictive biomarker for clinical response to ICI treatment. As is well known, high TMB levels are generally associated with improved tumor prognosis 40, 41. Furthermore, studies have indicated that mutations in TP53 and ARID1 in GC are averse to patient prognosis 42, 43. In this paper, we found that TIE1 expression was negatively correlated with TMB and positively correlated with the mutation of TP53 and ARID1, which means that TIE1 may hold promise as a novel biomarker for immunotherapy.

Biomarkers of GC can be used for early detection of tumors, tumor screening, diagnosis, tumor staging, monitoring the efficacy of surgery, chemotherapy, and radiotherapy for tumor patients, and prognostic assessment of tumors. Therefore, the main priority is identifying a novel biomarker to guide the diagnosis and treatment of GC. In this study, we detected the expression of TIE1 is significantly increased in GC patients compared to that of normal controls, ROC curve analysis demonstrated that the detection of TIE1 exhibited good sensitivity and specificity as a marker for GC. However, this study can only explain the detection of TIE1 playing a role in the diagnosis of GC, more large-scale studies and multi-center trials are still needed to confirm its advantages. Finally, it is expected that TIE1 can become a new biomarker for diagnosis of GC, assessment of progress, treatment effect, prognosis, and chemotherapy-acquired drug resistance soon.

## Conclusion

In summary, our findings indicate that TIE1 is significantly upregulated in GC, and its high expression means a worse prognosis. This study provides evidence that TIE1 may influence dysregulated immune cell infiltration within the TME. Specifically, we observed that TIE1 promotes tumor progression by upregulating immunosuppressive cells such as M2 while downregulating mast cell expression. Additionally, our results demonstrate a correlation between TIE1 expression and both ICIs and TMB, thereby highlighting the potential use of TIE1 as a guiding biomarker for immunotherapy in GC.

## Supplementary Material

Supplementary figure and tables.
