# Supplementary material for: Identification of the Expression of TIE1 and Its Mediated Immunosuppression in Gastric Cancer
Source: J Cancer. 2024 Mar 31;15(10):2994–3009. doi: 10.7150/jca.90891 (PMC11064258; doi:10.7150/jca.90891)
Supplement: Supplementary file 1 — Supplementary figure and tables. [file jcav15p2994s1.zip › Supplementary materials/supplementary figure.pdf]

IHC

Patients	Area	Mean	IntDen	Area%
1N	274951	0.61	167758.475	7.985
1N	347696	0.587	204012.182	10.098
1N	258292	0.644	166402.266	7.502
1N	284140	0.625	177452.75	8.252
1N	221138	0.664	146831.593	6.422
2N	538563	0.626	337146.631	18.503
2N	351061	0.701	246102.424	12.061
2N	520632	0.62	322875.058	17.887
2N	388228	0.68	263834.85	13.338
2N	200773	0.634	127342.442	6.898
3N	674647	0.625	421426.039	23.178
3N	552215	0.623	344293.101	18.972
3N	336921	0.614	206727.352	11.575
3N	213500	0.639	136334.288	11.19
3N	798721	0.669	534302.635	27.441
4N	311282	0.686	213603.5	10.694
4N	352637	0.585	206329.675	12.115
4N	537828	0.627	337111.21	18.477
4N	315754	0.65	205085.191	10.848
4N	488075	0.576	281334.897	16.768
5N	488357	0.68	332057.774	14.183
5N	571460	0.666	380360.418	16.597
5N	391189	0.67	262227.144	11.361
5N	948534	0.608	576688.272	27.548
5N	613336	0.624	382655.725	17.813
6N	293558	0.696	204194.912	10.085
6N	444968	0.584	259751.563	15.287
6N	303905	0.609	185014.829	10.441
6N	307758	0.706	217236.765	10.573
6N	353995	0.584	206860.155	12.162
7N	475252	0.602	286044.752	16.328
7N	501098	0.647	324226.268	17.216
7N	437083	0.611	266905.655	15.016
7N	251722	0.62	156128.804	10.114
7N	218258	0.637	139004.894	9.794
8N	184043	0.605	111425.951	6.323
8N	230813	0.596	137470.406	7.93
8N	307940	0.615	189327.456	10.58
8N	235685	0.712	167825.764	8.097
8N	275448	0.705	194191.637	9.463
9N	423634	0.663	280839.251	14.554
9N	280903	0.568	159655.821	9.651
9N	394358	0.609	240040.767	13.548
9N	260712	0.573	149456.528	8.957
9N	223421	0.564	126031.868	7.676
10N	350422	0.7	245454.799	12.039
10N	417120	0.682	284598.52	14.33
10N	367686	0.645	237109.689	12.632
10N	785809	0.63	494675.229	22.822
10N	941034	0.675	634846.803	27.33

Patients	Area	Mean	IntDen	Area%
1T	439191	0.563	247093.698	12.755
1T	339422	0.699	237241.826	9.858
1T	255555	0.72	183956.368	7.422
1T	303094	0.679	205892.386	8.803
1T	188589	0.6	113239.225	5.477
2T	324098	0.673	217963.077	9.413
2T	232666	0.692	160908.203	6.757
2T	243873	0.688	167729.586	7.083
2T	272692	0.662	180437.85	7.92
2T	230951	0.692	159848.497	6.707
3T	369058	0.628	231691.977	12.679
3T	206643	0.699	144340.909	7.099
3T	292473	0.67	195882.303	10.048
3T	200180	0.711	142309.653	5.814
3T	179443	0.756	135734.123	5.212
4T	414468	0.695	287967.558	14.239
4T	349511	0.711	248658.16	12.008
4T	592079	0.658	389819.295	20.341
4T	706979	0.679	480195.114	24.289
4T	398879	0.687	274157.891	13.704
5T	270882	0.675	182835.299	9.306
5T	450705	0.66	297612.782	15.484
5T	296196	0.693	205251.486	10.176
5T	472191	0.645	304583.439	16.222
5T	538058	0.682	367141.102	18.485
6T	470150	0.642	301620.676	16.152
6T	368983	0.629	232167.67	12.677
6T	287999	0.73	210224.043	9.894
6T	284168	0.744	211313.666	9.763
6T	417775	0.676	282533.08	14.353
7T	331922	0.641	212798.489	11.403
7T	484629	0.67	324556.421	16.65
7T	321990	0.646	208094.209	11.062
7T	509228	0.657	334447.108	17.495
7T	603010	0.56	337645.931	20.717
8T	478200	0.635	303660.959	16.429
8T	423721	0.64	271096.524	14.557
8T	447834	0.694	310914.734	15.386
8T	497919	0.705	350944.989	17.106
8T	417450	0.652	272131.594	14.342
9T	297183	0.774	230159.12	10.21
9T	399336	0.617	246556.817	13.719
9T	531129	0.622	330348.874	18.247
9T	413921	0.591	244754.418	14.221
9T	412722	0.653	269701.164	14.179
10T	349505	0.675	235963.122	12.008
10T	392551	0.668	262265.562	13.486
10T	546563	0.697	380832.422	18.778
10T	543796	0.694	377277.675	18.683
10T	299580	0.616	184422.628	10.292

	TIE1 expression		
Characteristics	High (n = 159)	Low (n = 160)	P-value
Age			0.401
≤65	74	67	
>50	85	93	
Gender			0.851
Female	59	61	
Male	100	99	
Grade			0.002
G1	5	2	
G2	40	70	
G3	114	88	
Stage			0.006
I	12	32	
II	58	43	
III	74	65	
IV	15	20	
T			0.024
T1	3	13	
T2	27	37	
T3	81	71	
T4	48	39	
N			0.282
N0	48	53	
N1	44	39	
N2	29	40	
N3	38	28	
M			0.385
M0	150	147	
M1	9	13	
